# Photo-and Heat-Induced Dismantlable Adhesion Interfaces Prepared by Layer-by-Layer Deposition

**DOI:** 10.1021/acs.langmuir.2c03233

**Published:** 2023-02-07

**Authors:** Miho Aizawa, Haruhisa Akiyama, Takahiro Yamamoto, Yoko Matsuzawa

**Affiliations:** † Research Institute for Sustainable Chemistry, National Institute of Advanced Industrial Science and Technology, Central 5, 1-1-1 Higashi, Tsukuba, Ibaraki 305-8565, Japan; ‡ Laboratory for Chemistry and Life Science, Institute of Innovative Research, Tokyo Institute of Technology, R1-12, 4259 Nagatsuta, Midori-ku, Yokohama 226-8503, Japan; § Department of Chemical Science and Engineering, Tokyo Institute of Technology, 2-12-1 Ookayama, Meguro-ku, Tokyo 152-8552, Japan; ∥ PRESTO, JST, 4-1-8 Honcho, Kawaguchi 332-0012, Japan; ⊥ Nanomaterials Research Institute, National Institute of Advanced Industrial Science and Technology, Central 5, 1-1-1 Higashi, Tsukuba, Ibaraki 305-8565, Japan

## Abstract

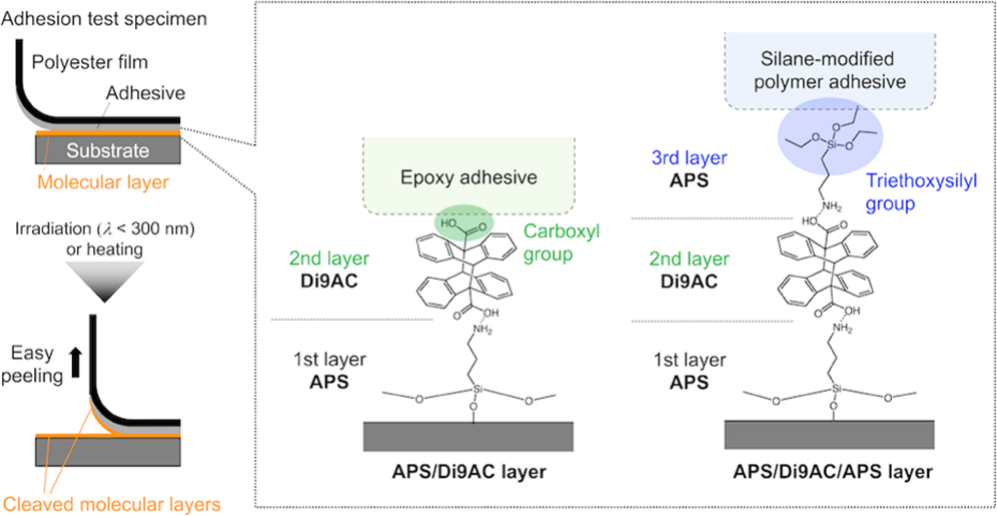

The development of a dismantlable adhesion technology that allows switching between bonding and debonding states using external stimuli is important for realizing renewable and sustainable material cycles. Controlling the adhesion interface is an effective approach to manipulate the adhesion strength; however, research on dismantlable systems focusing on the interface has not been proceeded. Recently, we demonstrated a novel dismantlable system based on a stimuli-responsive molecular layer comprising cleavable anthracene dimers, which strengthen the initial adhesive force by forming chemical bonds between the substrate and adhesive and can be dismantled when required via stimulation-induced bond breaking. Here, we evaluate the use of the anthracene-based molecular layer with different components for verifying its versatility in the adhesive/dismantling system. The formation of the cleavable molecular layer by the stacking of relevant molecules enabled its usage with two types of adhesives, an epoxy adhesive and a silane-modified polymer adhesive. The initial adhesive strengths were improved in both types of molecular layers by creating chemical bonds at the adhesion interfaces. Light irradiation or heating stimuli for 1 min reduced the peel strength by up to 65%, and dismantling occurred in the cleavable photodimer layer. This study expands the versatile applicability of the molecular layer-based dismantling system.

## Introduction

Adhesion is an important process in the production of a range of products using bonding materials.^1,2^ With the rapid increase in awareness on mitigating global pollution in recent years, there is an increasing demand for features in adhesion technologies that not only facilitate strong adhesion of target materials but also enable renewable and sustainable material cycling.^3^ An effective way of meeting this demand is to develop dismantlable adhesives that can be easily debonded using external stimuli as they facilitate higher-value outputs by allowing recycling or repair.^4−7^ Although many dismantlable adhesives have been realized by exploiting different types of stimuli, such as heat,^8−11^ light,^12−16^ electric field,^17^ magnetic field,^18^ and chemical treatment,^19,20^ the dismantlable adhesion technology is not widely available at present. The debonding mechanisms of dismantlable adhesives are mainly based on changes in the bulk properties of the adhesive, making it difficult to achieve both strong bonding and easy debonding by controlling the cohesiveness of the adhesive.

Another approach to control the adhesion strength is to treat the surface of the adherend with a focus on the state of the adhesion interface.^21^ One typical process involves surface treatment with a primer, which has been conventionally used to efficiently improve adhesion strength between materials.^22−24^ The related studies have indicated that thin layers formed at a molecular level by coating processes can influence adhesion strength. At the adhesion interface, physical or chemical bonds (such as hydrogen bonds or covalent bonds) have been suspected to play an important role in high-strength adhesion because of the strong bonding provided by such linkages.^1^ Hence, it is possible to realize both strong adhesion and easy separation by switching the chemical bonding at the adhesion interface.^25,26^ However, because of the difficulty in controlling the formation and breakage of chemical bonds, the progress in studies aimed at changing the chemical bonding state at the interface to reduce adhesion strength has been slow.

We previously developed a system that could be dismantled from the adhesion interface using a stimuli-responsive molecular layer consisting of chemical bonds that could be cleaved by applying a stimulus.^27,28^ In the molecular layer, the chemical bonding was controlled using anthracene molecules, which are known to undergo photodimerization and thermal/photocleavage reactions under external stimuli.^29,30^ When heat or light is applied to the cleavable molecular layer formed at the interface between the substrate and adhesive, the adhesion strength decreases, and separation occurs at the molecular layer.^28^ In addition, this molecular layer not only functions as a dismantlable system but also acts as a primer that increases the adhesion strength in the initial state. This function is achieved by the chemical bond formation between the molecular layer and the reactive functional groups of both the substrate and the adhesive. Thus, designing reactive functional groups in the molecular layer is important to realize the concept of this system.

**Figure 1:**
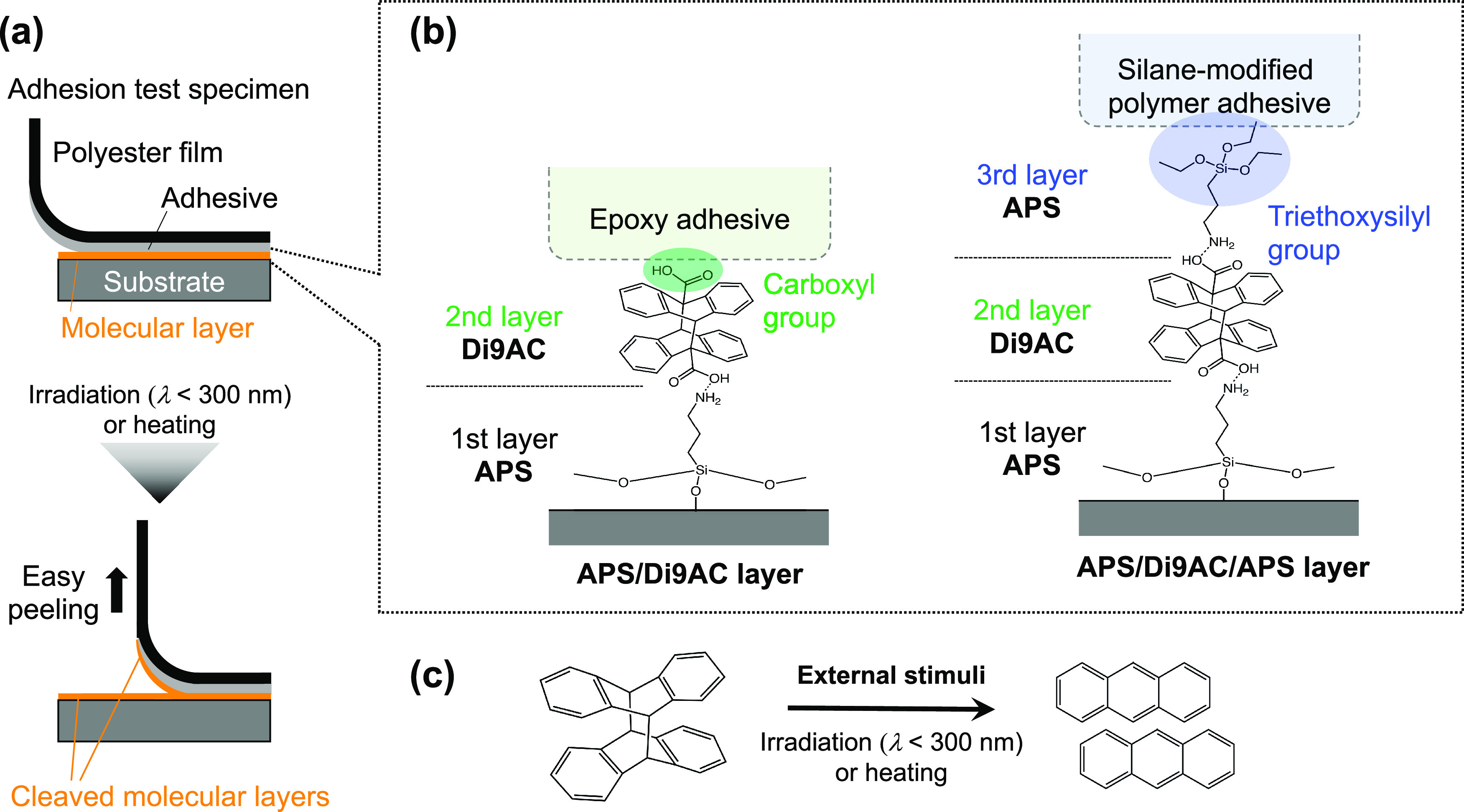
Overview of the molecular layer-based dismantlable adhesion system using a thermo/photocleavable molecular layer. (a) Conceptual illustration of a test specimen under 90° peeling test after the application of heat or light stimulus. Under the stimulus, the test specimen easily peels at the cleaved molecular layer. (b) Schematic of the constitution of the stacked molecular layer. Two types of molecular layers with different functional groups at the outermost surface were prepared based on the reactivity of the adhesive components. (c) Cleavage reaction of the anthracene photodimers into anthracene monomers.

Recently, we reported the successful preparation of cleavable molecular layers through acid–base interaction, which was inspired by layer-by-layer deposition.^31^ This process enables changes in the molecular layer composition according to the desired purpose, such as the presence of cleavable units and the selectivity of the functional group at the outermost surface. In this study, we investigated the effect of the composition of the molecular layer on a molecular layer-based dismantling system to explore the versatility of this system. Using molecular layers with or without the cleavable unit of the anthracene photodimer, we verified the concept of the dismantling system based on the chemical bond change. In addition, the functional group at the outermost surface of the molecular layer was changed by controlling the stacking procedure of 9-anthracenecarboxylic acid dimer (Di9AC) and 3-aminopropyltriethoxysilane (APS). We prepared two types of molecular layers containing carboxyl groups or triethoxysilyl groups at the outermost surface, which enables its reaction with different types of adhesive components (Figure 1). We successfully demonstrated stimuli-induced dismantling from the adhesion interface, even with a molecular layer prepared using simple acid–base interactions. This study indicates that molecular layer-based dismantling is a versatile technology that can take advantage of the characteristics of adhesives.

## Experimental Section

### Materials

9-anthracenecarboxylic acid (9AC) and APS were procured from Tokyo Chemical Industry Co., Ltd. Other reagents and solvents were purchased from Tokyo Chemical Industry Co., Ltd., Wako Pure Chemical Industry Ltd., or Kanto Chemical Co., Inc. and used without further purification unless otherwise stated. Di9AC was synthesized according to a published procedure.^31^

### Cleaning of the Substrates

Quartz substrates purchased from iTEC Science Co. Ltd. were cleaned as reported previously after cutting them into 30 × 10 mm^2^ pieces.^27^ The cut quartz substrates were cleaned ultrasonically in two consecutive steps using a 2.0% neutral detergent (Extran MA-02) solution in water and pure deionized water (Milli-Q grade, 18 MΩ·cm). Subsequently, they were immersed in an alkali detergent consisting of a 10 wt % solution of KOH (purity, 85.0%) in ethanol for 1 d. The substrates were then rinsed four times with deionized water through sonication for 20 min. Before use, the cleaned substrates were dried under vacuum for 1 h to ensure the removal of the residual adsorbed water.

### Molecular Layer Deposition

Dismantlable molecular layers were formed on the cleaned quartz substrates according to a previously reported method.^31^ First, the cleaned and completely dried substrates were immersed in an APS solution (1 wt %, dry toluene) for 1 h at 25 °C for aminosilylation. They were subsequently washed with dry toluene, heated at 100 °C for 30 min, sonicated in toluene for 1 min, and then dried under ambient conditions to obtain the substrate covered with the molecular layer consisting of APS molecules (APS layer). Next, the aminosilylated substrates were immersed in a Di9AC solution (0.1 mmol/L, acetone) for 30 min at 40 °C in a shaking bath. Thereafter, the substrates were removed gently and dried for 30 min at 80 °C. The substrates covered with the molecular layer consisting of APS and Di9AC (APS/Di9AC layer) were finally rinsed with acetone and dried under ambient conditions. Substrates covered with a molecular layer of 9AC (APS/9AC) were prepared using a similar process. For stacking APS molecules onto the Di9AC molecules, the substrates covered with the APS/Di9AC layer were immersed in an APS solution (0.1 mmol/L, chloroform) for 30 min at 40 °C in a shaking bath, removed gently, and dried for 30 min at 80 °C. Finally, the substrates with the stacked molecular layer (APS/Di9AC/APS) were rinsed with acetone and dried under ambient conditions.

### Photo/Thermo-Cleavage Reactions

For thermal cleavage of the molecular layer, the substrate was heated using a Mettler FP82HT (Tokyo, Japan). Light irradiation for photocleavage was performed using a light-emitting diode (CCS Inc. AC8375-280, λ = 280 nm). The light intensity was controlled to be 10 mW/cm^2^.

### Characterization Methods

Ultraviolet–visible (UV–vis) absorption spectra were measured to investigate the progress of the cleavage reaction using a JASCO V-670 spectrometer. Fluorescence spectra were recorded in the solid-state configuration using a JASCO FP-8550 spectrometer. Differential scanning calorimetry (DSC) was conducted at a heating and cooling rate of 10 °C/min using a HITACHI DSC 7000X.

### Evaluation of the Peel Strength

Quartz substrates (30 × 10 mm^2^) with the deposited molecular layer and cleaned bare quartz substrates (without a molecular layer) were used in this evaluation. The bare substrate was cleaned as described above. The specimens for peel strength measurements were prepared according to a previous report.^28^ Typically, the substrate was adhered to a hydrophilic polyester film (3M Japan Ltd. 9901P, thickness of 100 μm) using an adhesive. Two types of adhesives (Konishi Co., Ltd. MOS8 and ThreeBond Co. Ltd. TB1530-150) were selected depending on the composition of the molecular layer. The thickness of the adhesive layer was adjusted to be 100 μm. To complete the curing of the adhesive, the adhered specimens were maintained at 25 °C in an environment with a humidity of >50% for 24 h. After this process, a stimulus such as light irradiation or heating was applied as required from the quartz substrate side. The quartz substrates bonded with polyester films were adhered to a glass slide to fix them to the tensile test machine. The adhesion tests were performed at 25 °C using a tensile test machine (A&D Co., Ltd. STB-1225L, RTI-1310) equipped with an accessory for 90° peel tests (A&D Co., Ltd. J-PZ10-1kN). The peeling rate in the 90° peel test was 50 mm/min. The unit of peel strength was N/10 mm, based on the width of the specimens. The mean value of five replicate samples (*n* = 5) is reported as the peel strength.

## Results and Discussion

### Cleavage Reaction Conditions in the Molecular Layer

**Figure 2:**
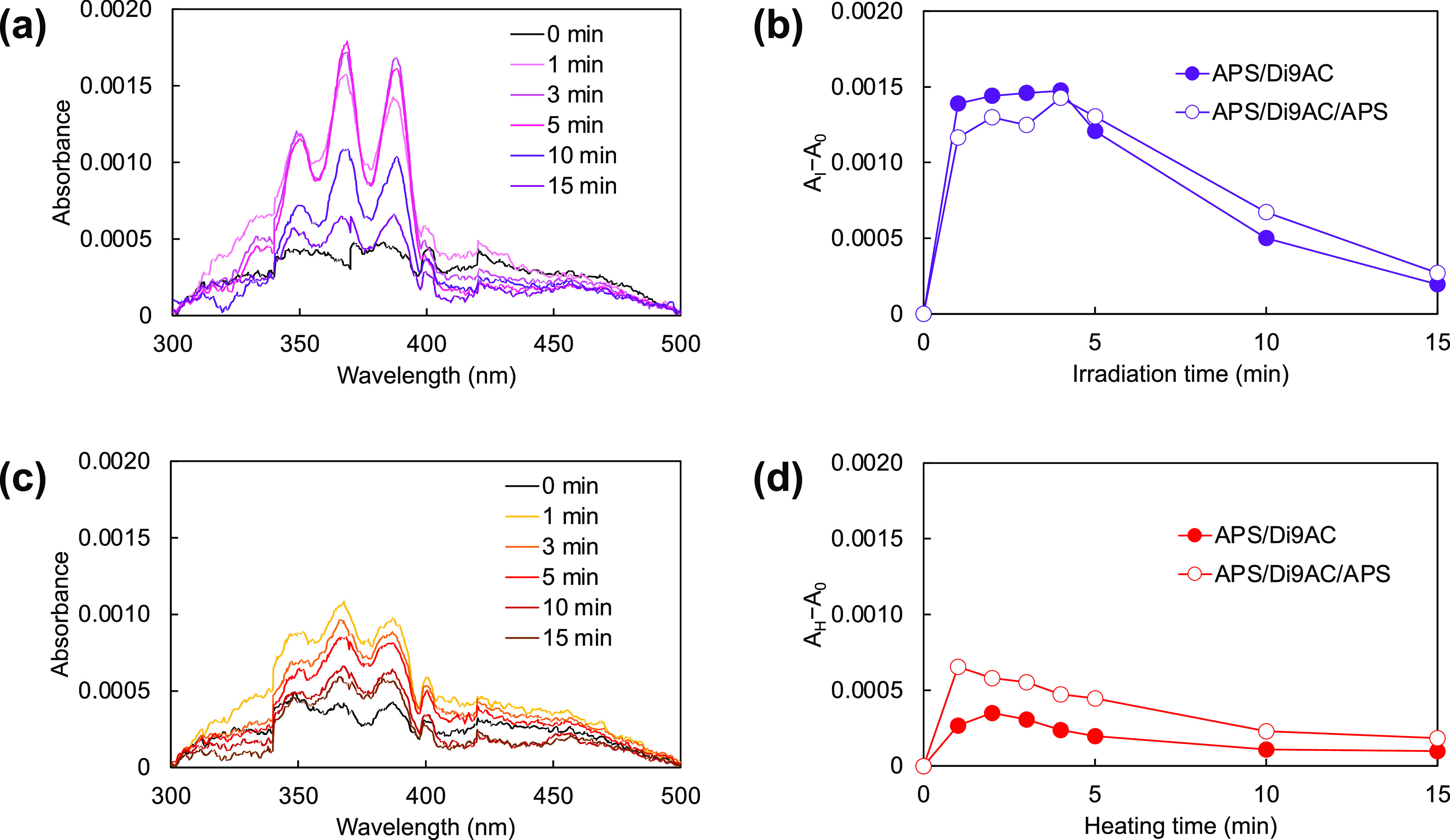
Evaluation of the progress of the cleavage reaction of the anthracene dimer via UV–vis absorption spectroscopy. (a) UV–vis absorption spectra of the APS/Di9AC/APS layer showing the appearance of regenerated monomers over time upon irradiating with 280 nm light. (b) Absorbance changes at 366 nm in the photocleavage reaction of two types of molecular layers; APS/Di9AC/APS and APS/Di9AC. *A*_I_ and *A*_0_ represent the absorbance at 366 nm after a given irradiation time and before irradiation, respectively. (c) UV–vis absorption spectra of the APS/Di9AC/APS layer showing the appearance of regenerated monomers over time upon heating at 180 °C. (d) Absorbance changes at 366 nm during the thermal cleavage reaction of the two types of molecular layers. *A*_H_ and *A*_0_ represent the absorbance at 366 nm after a given heating time and before heating, respectively.

A cleavable molecular layer was formed on a quartz substrate by stacking functional molecules (see Figure 1), and the cleavage reaction conditions were investigated to determine the applied stimuli for inducing the dismantling system in the adhesive state. The acid–base interaction used in the stacking process is widely applied in the formation of self-assembled monolayers.^32−34^ We prepared two types of cleavable molecular layer (APS/Di9AC layer and APS/Di9AC/APS layer) on a quartz substrate by stacking molecules based on the layer-by-layer process. The amino groups in APS units interacted with the carboxyl groups of the Di9AC layer via acid–base–type reaction (see Figure 1b), leading to the formation of multimolecular layers. If the molecular layer is formed as intended, the cleavage reaction of Di9AC should proceed upon light irradiation or heating, resulting in the appearance of an absorption peak derived from the anthracene monomer in the wavelength range of 350–400 nm.^35^ First, the photocleavage reactions of the molecular layers were investigated by irradiating the substrate with short-wavelength UV light. The appearance of the characteristic absorption peaks of the monomer confirmed the progress of the photo-induced cleavage reaction over several minutes (Figure 2a). However, the absorbance of the characteristic peak becomes smaller as the UV light irradiation time increases (Figure 2b). This phenomenon has also been confirmed in our previous study,^27^ which determined the generation of byproducts induced by the photoirradiation process. Next, to study the heating condition in the thermal cleavage reaction, changes in the UV–vis absorption properties of the substrates coated with the molecular layer of APS/Di9AC layer or APS/Di9AC/APS layer were monitored under heating at 180 °C. As shown in Figure 2c, the characteristic absorption peak of the monomeric anthracene appeared upon heating, indicating that the anthracene dimer in the molecular layers thermally cleaved into monomers. The heating time corresponding to the maximum absorbance of the monomer was different depending on the heating temperature, i.e., 1 min at 180 °C, 10 min at 160 °C, and 90 min at 140 °C (Figures 2d and S1). We determined that the anthracene dimer could be cleaved in a short duration by heating it at higher temperature. However, a decreasing trend in the absorbance due to the sublimation of the anthracene molecules is detected with continued heating, as the same tendency as in the previous study.^31^

**Figure 3:**
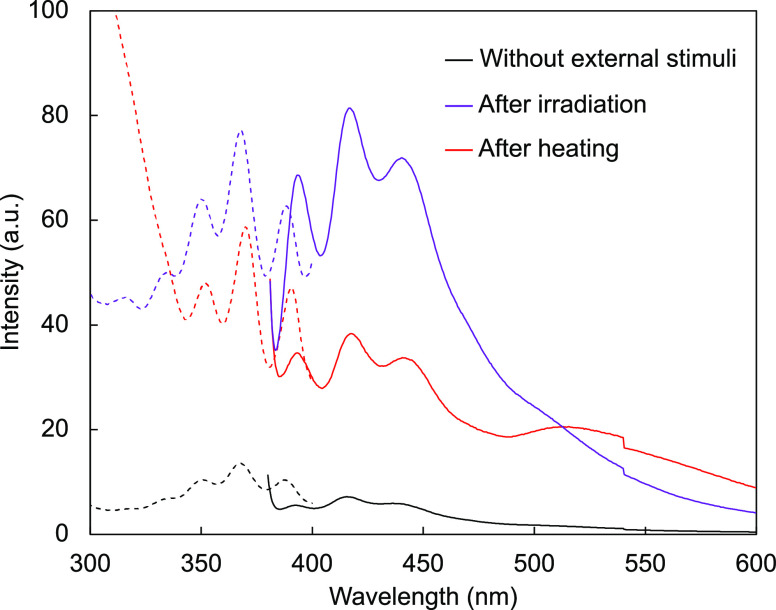
Fluorescence excitation (left, dashed line) and emission (right, solid line) spectra of APS/Di9AC layer before and after stimulation. The excitation and emission wavelength were 365 and 420 nm, respectively.

In addition, the photocleavage reaction was monitored using fluorescence spectroscopy. Figure 3 shows the excitation and emission spectra of the APS/Di9AC layer before and after photoirradiation with 280 nm wavelength light for 1 min or heating at 180 °C for 1 min. In the results of after stimulation, the characteristic fluorescence peaks were detected in both the emission and excitation spectra. The peak shape and the fluorescence maximum were almost the same as a molecular layer consisting of an anthracene monomer (APS/9AC layer) (Figure S2). This implies that photoirradiation and heating of the APS/Di9AC layers helped the cleavage reaction to proceed, and the produced anthracene monomers were stacked monomerically on the substrate.

### Effect of Applying Stimuli on Adhesion Tests Using Epoxy Adhesives

To investigate the effect of the stimulation for causing the dismantling system on the adhesion test, we conducted 90° peel tests using bare substrates, which have no molecular layer. We conducted peel tests on the specimens composed of the substrate without molecular layer to evaluate the effect of the stimuli on the bulk states of the adhesives. The peel strength is known to reflect the state of the adhesive/adherend interface, and a rigid specimen bonded with a flexible substrate on at least one side is used to measure the peel strength.^23,24^ The test specimen is prepared by adhering a quartz substrate and a flexible polyester film, as illustrated in Figure 1a. The peel test is conducted by pulling off the adhered polyester film in the orthogonal direction to the quartz substrate. The peel strength was calculated from the middle range of the measured tensile force graph that reaches a plateau in the peeling process to eliminate the effect of the influence of the adhesion edge of the test specimen.

Based on the results of the cleavage reaction of the APS/Di9AC molecular layer (Figure 2), the applied stimuli were selected as 280 nm light irradiation for 1 min or 180 °C heating for 1 min. As shown in Figure S3, no significant difference in the peel strength was observed with a change in the stimulus. However, upon comparing the physical states of the peeled specimens, the adhesive remained on the entire surface of the substrate only in the case of the heated sample. As the adhesive was discolored, we inferred that heating at 180 °C degraded the adhesive component, even if the heating time was only 1 min. Therefore, we investigated the effect of heating at a lower temperature. The thermal cleavage reaction in the molecular layer proceeds upon heating for a long time at a lower temperature of 140 °C, as shown in Figure S1; then, the specimen was heated at 140 °C for 90 min. However, even in this case, the adhesive was discolored and remained on the detached substrate (Figure S3). The DSC measurement of the epoxy adhesive implied that the heating stimuli induced the decomposition of the adhesive components because a significant peak was observed above 100 °C in the first heating process (Figure S4). And also, the baseline shift was observed for both the heating and the cooling processes at 77.0 and 77.4 °C, respectively. These results indicate the heating stimulus is not suitable for inducing dismantlable adhesion with this epoxy adhesive.

### Effect of the Formation of the Molecular Layer at the Adhesion Interface

**Figure 4:**
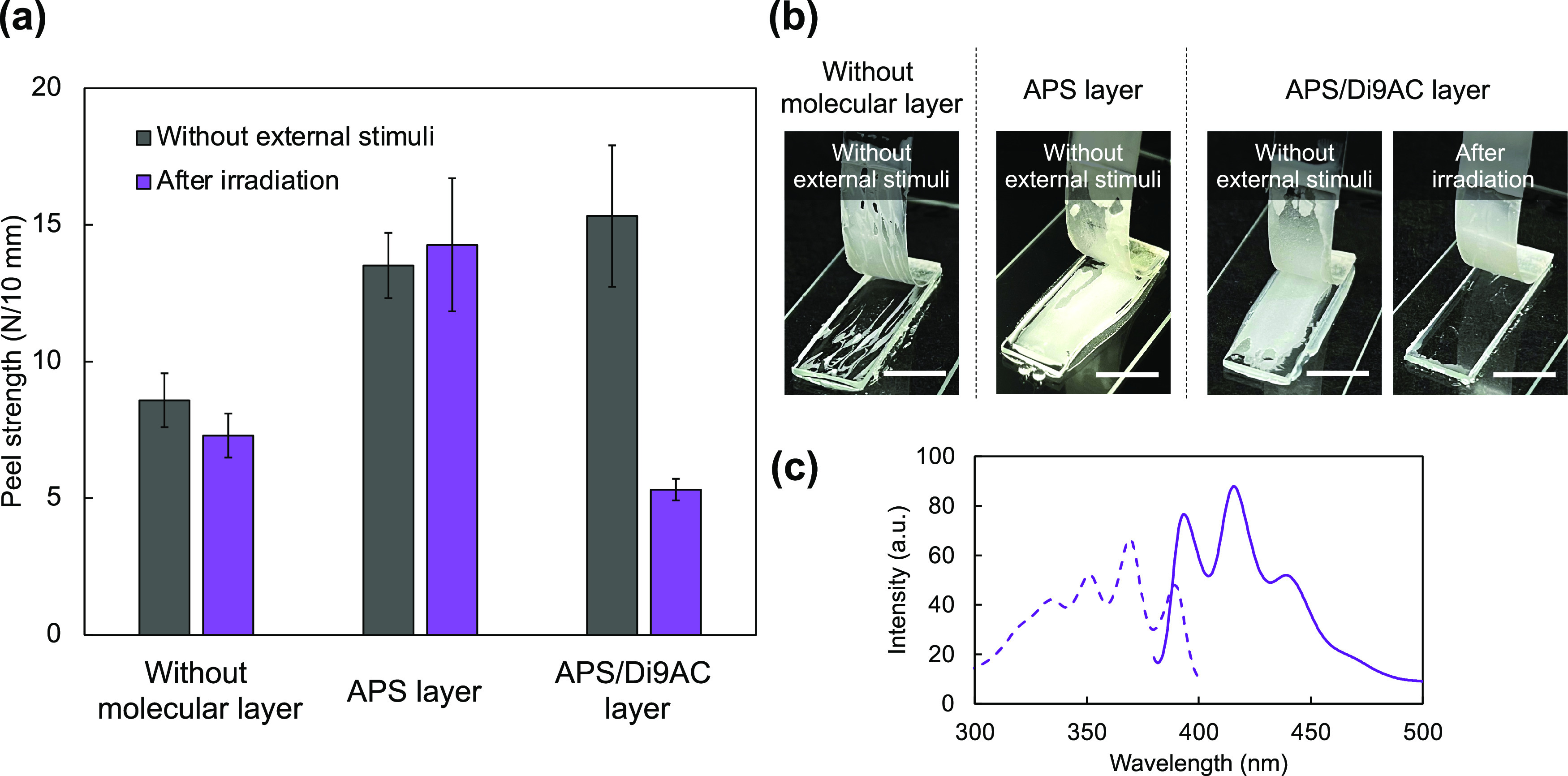
Effect of the APS layer and APS/Di9AC layer on the peel strength upon using an epoxy adhesive. (a) Average peel strengths in the extension range of 10–25 mm under different conditions (without any stimulus and after light irradiation). Error bars represent the standard deviation. (b) Photographs of specimens peeled under different conditions. Scale bars, 1 cm. (c) Fluorescence excitation (dashed line) and emission (solid line) spectra of quartz substrates covered with the APS/Di9AC layer after peeling tests following UV irradiation. The excitation and emission wavelengths were 365 nm and 420 nm, respectively.

Figure 4a presents the results of averaged peel strengths of the test specimen applied to each condition. To evaluate the effect of the cleavable unit on adhesion, two types of molecular layers were used: APS layer and APS/Di9AC layer. Initially, we compared the peel strengths of these molecular layer-covered samples without applying an external stimulus. The calculated peel strengths of both samples were almost the same and determined to be 15 N/10 mm, which is twice that of the uncoated control sample. This is strong evidence that the molecular layer at the interface affects the adhesion strength. Considering the organization of the molecular layers, amine groups and carboxyl groups were present at the outermost surface of APS layer and APS/Di9AC layer, respectively. It is known that amine groups are generally used for curing of epoxy resins, and esterification-induced cross-linking can take place between epoxide and carboxyl groups.^36−40^ Thus, it is reasonable that covalent bonds are created at the adhesion interface by these molecular layers. Note that the molecular layer prepared by layer-by-layer deposition includes acid–base interaction between the stacked molecules. The bond energy of hydrogen bonds and covalent bonds are around 2.5–125 and 200–800 kJ/mol, respectively.^41^ This implies that the acid–base interaction is weaker than the covalent bonds. In spite of this, we confirmed an improvement in the initial adhesive strength in the case of using APS/Di9AC layer. Therefore, it is reasonable that the bonding strength of the acid–base interaction is strong enough for this specimen.

### Dismantling Behavior Using the Cleavable Molecular Bilayer

The behavior of the dismantlable system was investigated by conducting peeling tests after the stimulation of the sample. In the case of the sample with the APS layer (without the cleavable unit), the light irradiation had no effect on the peel strength and the failure modes (Figure S5). In contrast, in the case of the test specimen prepared with the APS/Di9AC layer, the peel strength decreased significantly after the light irradiation (Figure S6), and the failure modes of the stimulated specimens also reflected the influence of the stimulation (Figure 4b). In the absence of the stimulus, the detachment of the flexible film from the substrate occurred within the body of the adhesive, and the adhesive residue remained on the substrate surface. In contrast, the test specimen stimulated by light irradiation showed no remaining adhesive on the substrate surface. These detachment behaviors are defined as a cohesive failure and interfacial failure, respectively.^42^ The summarized results of the calculated peel strength of the specimens adhered by the epoxy adhesive clearly showed peel strength dependence in the stimulation only in the case of APS/Di9AC layer (Figure 4a). In the case of APS/Di9AC layer, light irradiation resulted in a 65% reduction in the peel strength compared with the peel strength measured without any external stimulus.

Considering the principle of the dismantlable system consisting of a cleavable molecular layer, anthracene monomers generated under the applied stimuli are expected to remain on the quartz surface. Characteristic fluorescence bands of anthracene were detected by the spectroscopic measurement of the detached specimens after dismantling by light irradiation (Figure 4c). This proves that the cleaved anthracene monomers remained on the substrate surface after the flexible adherend was peeled off; that is, the detachment of the substrate from the adherend proceeded at the photodimer in the APS/Di9AC layer. These results indicate that the molecular layer formation process based on the acid–base interaction between APS and Di9AC is acceptable for working as a primer, and the existence of the cleavable molecules is the key to realizing the dismantling system.

### Adhesion and Dismantling Properties of APS/Di9AC/APS Molecular Layer

**Figure 5:**
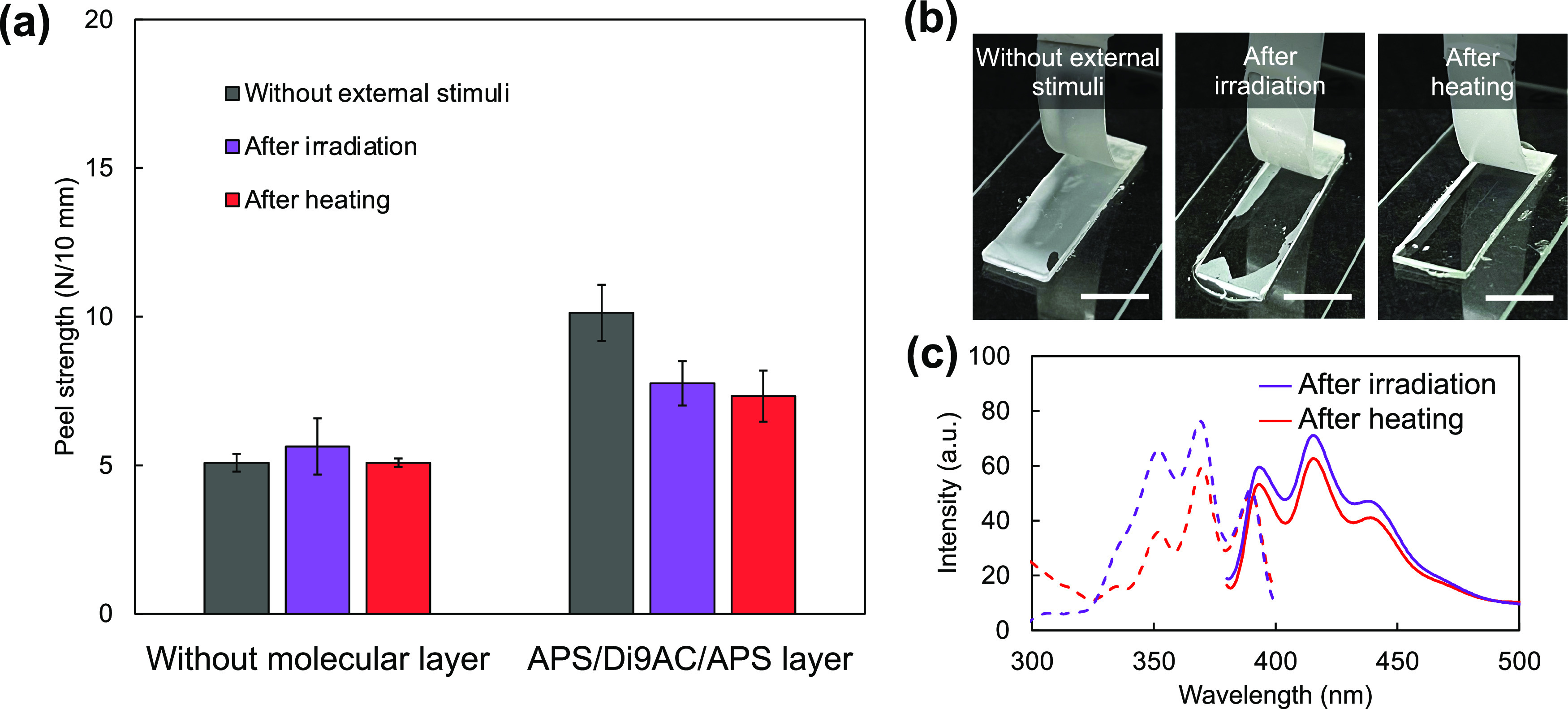
Effect of the APS/Di9AC/APS layer on the peel strength measured using a silane-modified polymer adhesive. (a) Average peel strengths in the extension range of 10–25 mm for different conditions (without any stimulus, after light irradiation, and after heating). Error bars represent the standard deviation. (b) Photographs of specimens with the APS/Di9AC/APS layer being peeled after processing under different conditions. Scale bars, 1 cm. (c) Fluorescence excitation (dashed line) and emission (solid line) spectra of quartz substrates covered with the APS/Di9AC/APS layer after peeling tests following UV irradiation and heating. The excitation and emission wavelength were 365 and 420 nm, respectively.

The cleavable molecular layer was implemented with different functional groups, making it possible to form chemical bonds with different types of adhesives. Therefore, we designed the molecular layer as an APS/Di9AC/APS layer, whose surface was covered by triethoxysilyl groups to react with the silyl groups of a silane-modified polymer adhesive. The peel tests of the specimens with and without the molecular layer were conducted and tensile force plots were obtained (Figures S7 and S8), and the calculated results are summarized in Figure 5a. To evaluate the effect of the cleavable molecular layer on adhesion, we compared the peel strengths of these samples without applying an external stimulus. The peel strength of the test specimen covered by the molecular layer was twice that of the uncoated control sample. In addition, comparing the photographs of the peeled specimens (Figures 5b and S7), the failure modes were different for the existence of the molecular layer: cohesive or interfacial failure occurring for the specimens with or without molecular layer, respectively. These results are strong evidence that the molecular layer at the interface affects the adhesion strength. Note that the composition of the APS/Di9AC/APS layer is almost the same and uses the same polymer adhesive as that reported previously.^28^ The average peel strength of the test specimen of the APS/Di9AC/APS layer was determined to be 10 N/10 mm, which is almost the same as that observed in our previous report.^28^

The dismantling properties of the APS/Di9AC/APS layer were evaluated by applying a stimulus to the specimen. The conditions of the applied stimuli were determined based on the cleavage characteristics of the anthracene dimer: irradiation with 280 nm light for 1 min or heating at 180 °C for 1 min. While the test specimens consisting of a substrate without a molecular layer showed the same peel strength before and after the stimulation, the peel strength of the test specimen covered with the multimolecular layer was significantly decreased in both cases of light irradiation and heating (Figure 5a). As compared with that of the sample peeled without external stimuli, the peel strength was reduced by 28 and 23% by heat treatment and light irradiation, respectively. In addition, both stimulated specimens showed clear substrate surfaces after the peeling test, indicating that the detachment progressed at the adhesive/adherend interface, as shown in Figure 5b. The fluorescence excitation and emission spectra showed the characteristic bands in the photo- or thermo-cleaved samples after the peeling tests, implying the existence of the cleaved anthracene molecules on the substrate surfaces (Figure 5c). These results proved that the concept of the dismantlable molecular layer can be used for the several-molecules-stacked molecular layer. However, the reduction rate of the peel strength was lower than that observed in our previous study.^28^ In this study, the coverage rate of the anthracene dimer, which is the key material responsible for dismantling, might be low because of the stacking procedure. Particularly, in the case of applying thermal stimuli, the specimen bonded tightly owing to the softening of the adhesive under heating. Therefore, the difference in the coverage rate of molecules due to changes in the molecular layer formation process possibly causes a decrease in the dismantling ability as compared with the previous study.^28^

Upon comparing the peel strength obtained using the epoxy adhesives and the silane-modified polymer adhesives (Figures 4a and 5a), the measured tensile force of the samples with the molecular layer was higher for the epoxy case. In both cases, the measured force reflects the cohesiveness of the adhesives because failure occurred within the body of the adhesive. This indicates that the strength of the interfacial adhesion due to the molecular layer was higher than the cohesive force of the epoxy adhesive. Further studies with other types of adhesives will allow a detailed assessment of the strength of the chemical bonds in the molecular layer in the adhered state.

## Conclusions

In this study, we investigated the performance of an anthracene-based cleavable molecular layer that facilitates strong adhesion and subsequent easy separation at the adhesion interface when desired. The bonding and debonding could be achieved based on the state of the chemical bonds at the adhesion interface. The concept of this multimolecular layer system was inspired by the basic theory of the layer-by-layer assembly developed for functionalizing various surfaces,^43−45^ and we successfully constructed the molecular layer by stacking appropriate functional molecules. We measured the peel strength of the substrate coated with the molecular layer and bonded to a flexible adherend using two types of adhesives: an epoxy-based adhesive and a silane-modified polymer adhesive. The molecular layers were designed individually for each adhesive, and in both cases, the strengthening and weakening of the adhesive force at the interface could be controlled using an external stimulus. One of the greatest strengths of this system is its ability to take advantage of the adhesive properties of functional adhesives such as the adhesion strength because this system only affects the adhesion interface. In fact, dismantling from the adhesion interface was observed regardless of the type of adhesive used. Moreover, the peel strength of the test specimen in the initial state reflected the cohesiveness of the adhesive. The results indicate that the molecular layer-based dismantling process is highly attractive for realizing renewable and sustainable material cycles.

## Supporting Information Available

Absorbance changes in UV–vis absorption spectra of molecular layers; fluorescence spectra of a molecular layer; peel strength measurements of specimens with and without molecular layer; and differential scanning calorimetry (DSC) thermograms of an adhesive (PDF)

## Abbreviations

Di9AC: 9-anthracenecarboxylic acid dimer
APS: 3-aminopropyltriethoxysilane
9AC: 9-anthracenecarboxylic acid
DSC: differential scanning calorimetry
