# Supplementary material for: Photo-and Heat-Induced Dismantlable Adhesion Interfaces Prepared by Layer-by-Layer Deposition
Source: Langmuir. 2023 Feb 7;39(7):2771–8. doi: 10.1021/acs.langmuir.2c03233 (PMC9948544; doi:10.1021/acs.langmuir.2c03233)
Supplement: Supplementary file 1 — la2c03233_si_001.pdf [file la2c03233_si_001.pdf]

Supporting Information

Photo-and Heat-induced Dismantlable Adhesion Interfaces Prepared by Layer-by-Layer Deposition

Miho Aizawa[*1,2,3,4], Haruhisa Akiyama*[5], Takahiro Yamamoto*[1], Yoko Matsuzawa*[*1]

[1]Research Institute for Sustainable Chemistry, National Institute of Advanced Industrial Science and Technology, Central 5, 1-1-1 Higashi, Tsukuba, Ibaraki 305-8565, Japan

[2]Laboratory for Chemistry and Life Science, Institute of Innovative Research, Tokyo Institute of Technology, R1-12, 4259 Nagatsuta, Midori-ku, Yokohama 226-8503, Japan

[3]Department of Chemical Science and Engineering, Tokyo Institute of Technology, 2-12-1 Ookayama, Meguro-ku, Tokyo 152-8552, Japan

[4]PRESTO, JST, 4-1-8 Honcho, Kawaguchi 332-0012, Japan

[5]Nanomaterials Research Institute, National Institute of Advanced Industrial Science and Technology, Central 5, 1-1-1 Higashi, Tsukuba, Ibaraki 305-8565, Japan

E-mail: aizawa@res.titech.ac.jp, yoko-matsuzawa@aist.go.jp

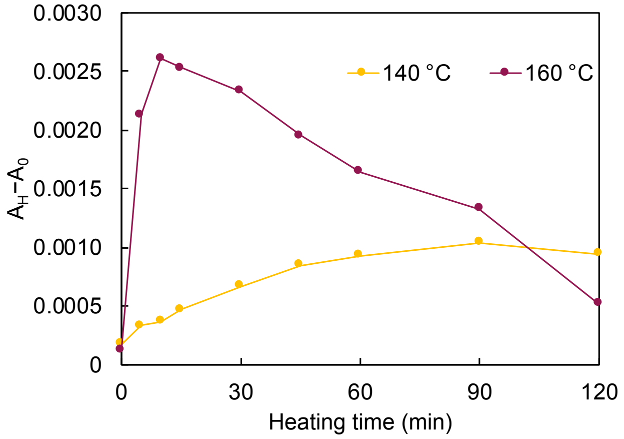

Figure S1. Absorbance changes at 366 nm in UV-vis absorption spectra of APS/Di9AC layer upon heating at 140 °C and 160 °C.

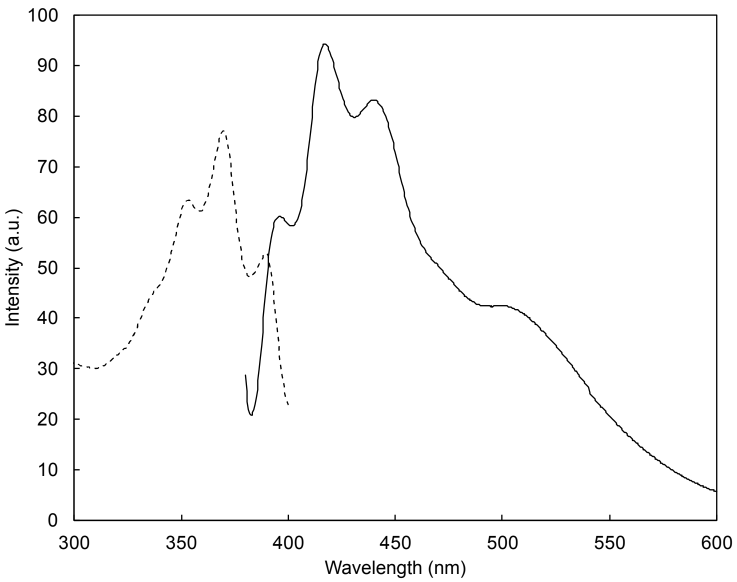

Figure S2. Fluorescence excitation (dashed line) and emission (solid line) spectra of APS/9AC layer. The excitation and emission wavelength were 365 nm and 420 nm, respectively.

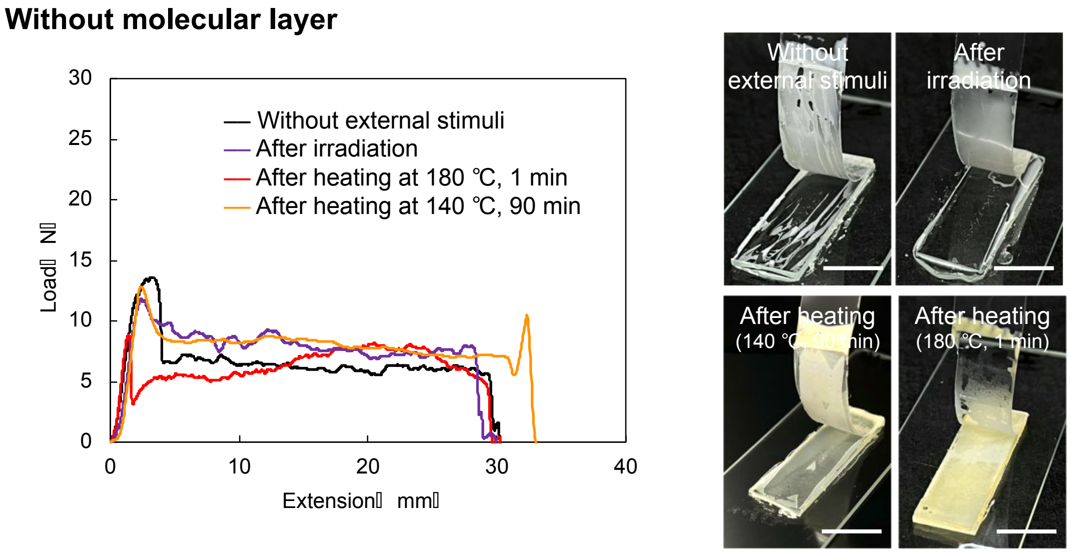

Figure S3. Peel strength measurements and photographs of specimens peeled under different conditions. Specimens were prepared substrates without molecular layer using epoxy adhesives. Scale bars, 1 cm.

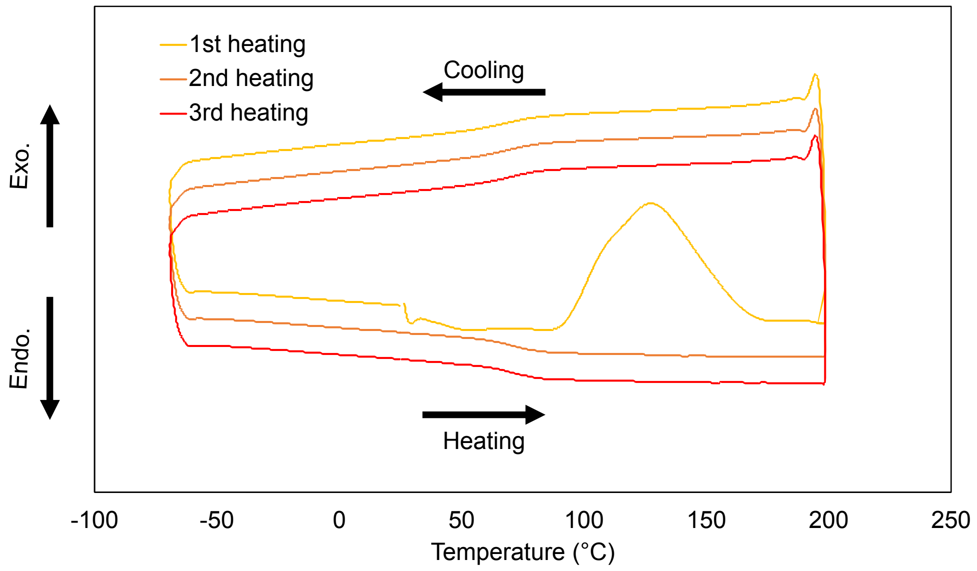

Figure S4. DSC thermograms of the epoxy adhesive, where the scanning rate of 10 °C/min.

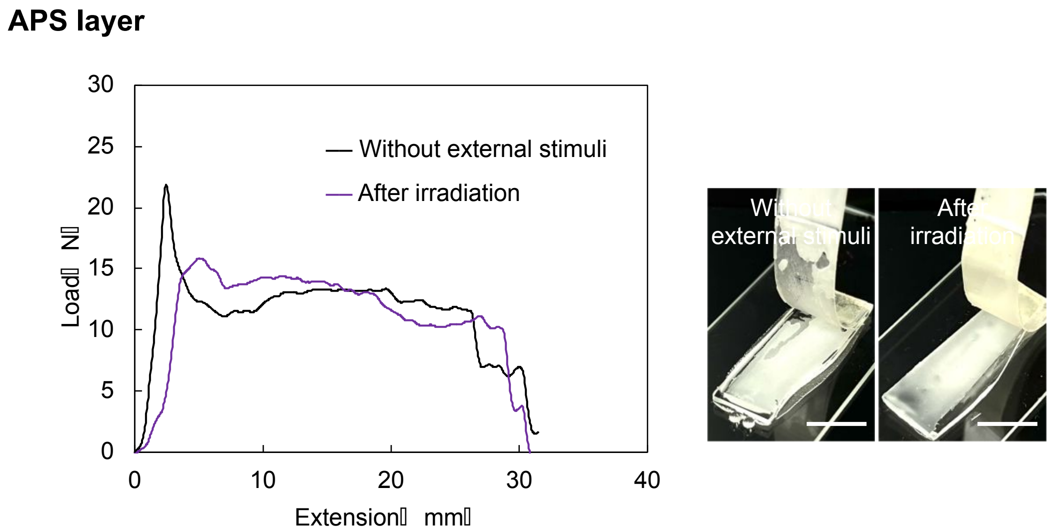

Figure S5. Peel strength measurements and photographs of specimens peeled under different conditions. Specimens were prepared substrates covered with APS layer using epoxy adhesives. Scale bars, 1 cm.

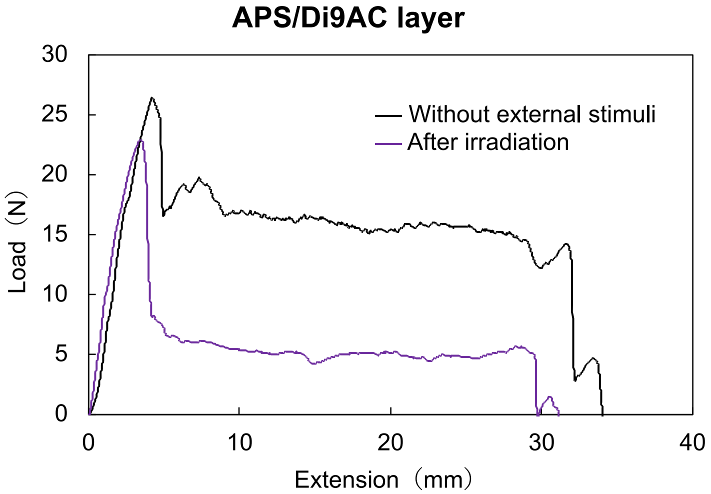

Figure S6. Peel strength measurements peeled under different conditions. Specimens were prepared substrates covered with APS/Di9AC layer using epoxy adhesives.

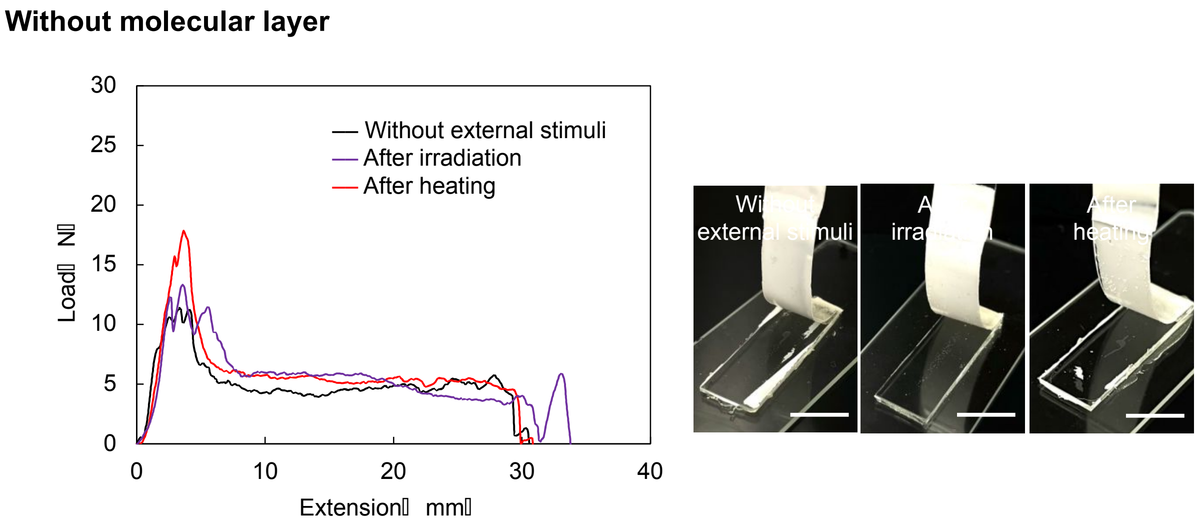

Figure S7. Peel strength measurements and photographs of specimens peeled under different

conditions. Specimens were prepared substrates without molecular layer using silane-modified polymer adhesives. Scale bars, 1 cm.

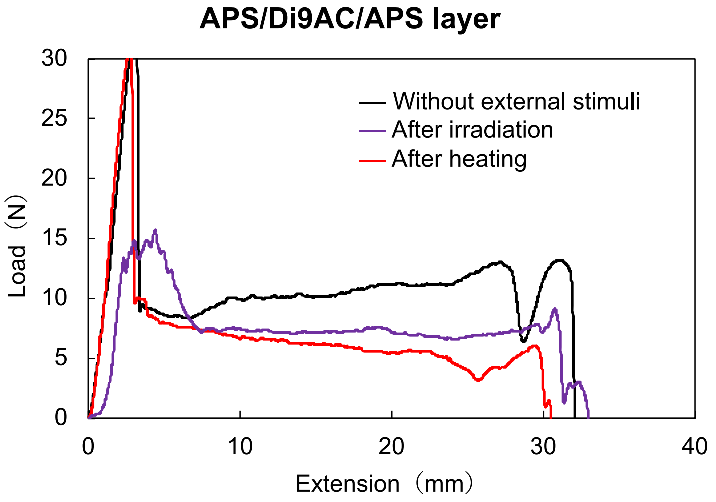

Figure S8. Peel strength measurements peeled under different conditions. Specimens were prepared substrates covered with APS/Di9AC/APS layer using silane-modified polymer adhesives.
